# Supplementary material for: Identification of Genes Associated with Gut Microbiota Metabolites in Type 2 Diabetes Mellitus Complicated by Acute Cerebral Infarction Based on Bioinformatics and Mendelian Randomization
Source: Biomedicines. 2026 Oct 17;14(10):2371. doi: 10.3390/biomedicines14102371 (PMC13702449; doi:10.3390/biomedicines14102371)
Supplement: Supplementary file 1 [file biomedicines-14-02371-s001.zip › Figure S3/genus.Sellimonas.id.14369 ACI.pdf]

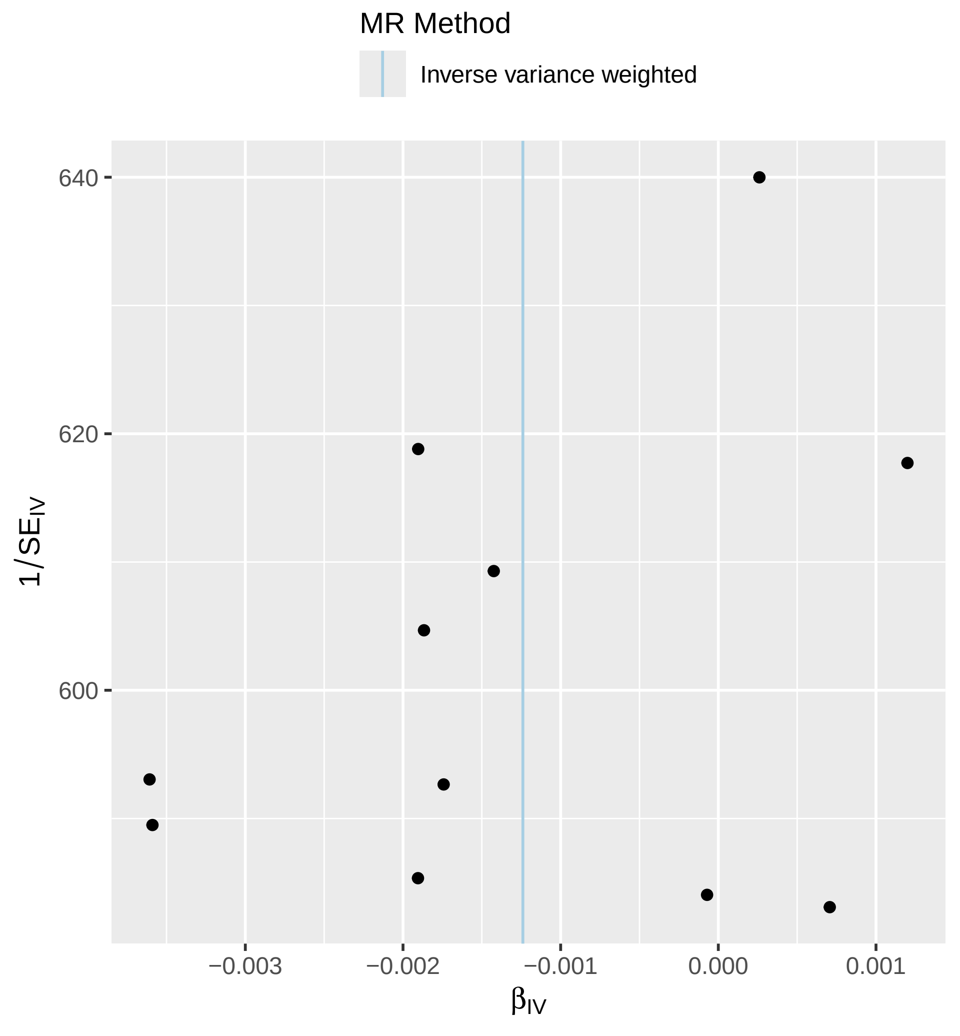

MR Method
Inverse variance weighted
640
620
600
1/SE_IV
−0.003
−0.002
−0.001
0.000
0.001
β_IV
